# Words and Worms: The Nosology of Strongyloidiasis

**DOI:** 10.4269/ajtmh.26-0113

**Published:** 2026-05-05

**Authors:** Christopher V. Radcliffe, Joseph M. Vinetz

**Affiliations:** Section of Infectious Diseases, Department of Medicine, Yale School of Medicine, New Haven, Connecticut, USA

## Abstract

Strongyloidiasis impacts millions of people on several continents. Although chronic infection is often subclinical, select forms of immune dysregulation predispose patients to severe clinical manifestations resulting from acceleration of the nematode’s autoinfective cycle. The terms “hyperinfection syndrome” and “disseminated strongyloidiasis” commonly refer to these disease states; however, contemporary usage is not uniform. We herein review the origins of terms related to the nosology of strongyloidiasis and highlight important mechanisms of disease pathogenesis that make untreated strongyloidiasis highly relevant to contemporary medicine.

## INTRODUCTION

Globally, *Strongyloides stercoralis* is estimated to impact hundreds of millions of people.^1^ Infection of the human host generally occurs after skin contact with soil contaminated with feces, and the nematode’s autoinfection cycle renders it capable of persisting for decades.^2^ In general, the life cycle of *S. stercoralis* has an environmental phase and a parasitic phase. The environmental phase begins when an infected human host defecates and deposits rhabditiform larvae into soil. Depending on environmental conditions, rhabditiform larvae may molt into infective filariform larvae that penetrate the skin of a human host and migrate to reach the small intestine. Alternatively, soil-dwelling rhabditiform (noninfective) larvae may become free-living adult worms that sexually reproduce eggs that give rise to more rhabditiform larvae, which may then become filariform larvae—the infective form. In the parasitic phase, filariform larvae migrate to the small intestine after penetrating skin. Larvae then mature into adult female worms that produce eggs that hatch into rhabditiform larvae. The ability of rhabditiform larvae to molt into filariform larvae inside the human host makes autoinfection possible because filariform larvae can directly reinfect the same human host by penetrating perianal skin and/or gastrointestinal mucosa.

In contemporary practice, strongyloidiasis refers to one of several possible disease states. Acute strongyloidiasis is uncommonly reported, but the nematode’s initial tissue migratory phase can cause symptoms such as diarrhea, cough, rash, and/or urticaria. Chronic strongyloidiasis is typically subclinical, but it can lead to gastrointestinal or multisystem symptoms of variable intensity with or without peripheral eosinophilia. The terms “hyperinfection” or “disseminated strongyloidiasis” refer to dramatic, severe clinical manifestations resulting from exuberant acceleration of the nematode’s autoinfection cycle.^2^^,^^3^ These manifestations can occur decades after an initial infection, and predisposing factors include glucocorticoid exposure, solid organ transplantation, and human T-cell leukemia virus-1 coinfection.^3^ Not all forms of immune suppression predispose to hyperinfection or disseminated strongyloidiasis, and it has been hypothesized that the strong association with glucocorticoids is due to steroid metabolites that structurally mimic nematode molting signals known as ecdysteroids.^4^ The high mortality of hyperinfection or dissemination and the rising prevalence of immunocompromised persons underscore the importance of untreated strongyloidiasis.^2^^,^^3^^,^^5^

For clinicians, understanding the convoluted natural history of strongyloidiasis is fundamental to its effective treatment. Imagine the hypothetical case of an adult from Southeast Asia who receives high-dose glucocorticoids as induction therapy for connective tissue disease and then develops septic shock due to *Escherichia coli* bacteremia. After anatomical causes (e.g., biliary stricture) and occult foci of infection are excluded, *S. stercoralis* hyperinfection or dissemination ought to be considered. For both disease states, the pathogenesis of *E. coli* bacteremia stems from bacterial translocation from gastrointestinal ulcerations produced by filariform larvae and/or filariform larvae carrying enteric flora as they migrate to extraintestinal sites. In both cases, larvae disrupt the normal gut–blood barrier. Before approaching contemporary definitions of hyperinfection and dissemination, it is instructive to consider the evolution of language related to strongyloidiasis.

The first modern description of strongyloidiasis in European literature occurred in 1876 when Normand microscopically examined the stools of French colonial troops who returned from Vietnam (Cochin-China) to France with acute diarrheal illness.^6^ Despite only imbibing imported water, Normand reportedly had “Cochin-China diarrhea” himself and later identified another form of the nematode at autopsy in a soldier who succumbed to the illness.^6^^,^^7^ Several years elapsed before investigators recognized that different larval forms and the adult worm belonged to the same organism’s life cycle. By the turn of the 20th century, certain cases of strongyloidiasis were found to have “enormous number of worms” in the small bowel, and “extraordinary multiplication” of *S. stercoralis* in the human host had been theorized as an explanation.^7^ A 1904 textbook describes Teissier’s 1895 report of “embryos” leading to fever after entering systemic circulation, but the author theorized that this represented filariasis and strongyloidiasis coinfection.^7^^,^^8^ The pathogenicity attributed to *S. stercoralis* varied.^6^^,^^7^

In a 1911 report, Gage described a fatal case of strongyloidiasis with fever, diarrhea, skin manifestations, and larval forms repeatedly identified in sputum.^9^ Apropos of Teissier’s report, blood was repeatedly examined without identification of larvae.^8^^,^^9^ At autopsy, the patient had adults, eggs, and larval forms in the bowel lumen with microscopic evidence of larvae invading the bowel wall. Based on larvae in sputum despite weeks of hospitalization, Gage hypothesized, “[t]hese patients must reinfect themselves” and posited a “vicious circle,” whereby larval forms reenter the skin or access the lymphatic system by traversing the bowel wall and eventually reach the lungs via the thoracic duct before being swallowed back into the gastrointestinal tract.

In the 1920s, Fülleborn introduced the term “autoinfection” when discussing strongyloidiasis and observations related to larval penetration of skin.^10^ It is not known whether Fülleborn knew of Gage’s case report because Gage had explicitly posited that larvae “were gaining entrance through the skin.”^6^^,^^9^ The term autoinfection gained currency when describing the life cycle of *S. stercoralis* in the human host. For example, a 1929 report of filariform larvae in the colonic submucosa and mesenteric lymph nodes at autopsy ascribed the patient’s death to strongyloidiasis and concluded that “autoreinfection takes place constantly.”^11^

Faust went on to use the term “hyperinfection” in 1930 when discussing what were interpreted to be “hyperinfective” strains of *S. stercoralis* in Panama that led to filariform larvae in the bowel lumen.^12^ Immediately thereafter, not all reports adopted the term “hyperinfection.” A 1936 report used the terms “severe fatal infestation” and “overwhelmingly extensive infestation” to describe a terminal strongyloidiasis case with abundant filariform larvae in the small bowel wall and meso-appendix as well as larvae in hepatic lymph vessels at autopsy.^13^ The authors commented on Faust’s experimental data on “hyperinfection” but used the terms “reinfection” or “internal autoinfestation” to describe their observations.^13^ Faust and de Groat discussed this 1936 case in their subsequent article and labeled it an “overwhelming internal invasion” before presenting their own case of “extensive hyperinfection.”^14^ They defined “hyperinfection” as internal autoinfection and differentiated this term from autoinfection due to larval forms entering skin. They went on to use a dizzying list of terms, including “reinfection,” “autoinfection of Fülleborn,” “hyperinfection,” and “self-infection,” in the same article.^14^

By the 1940s, some authors referred to the “mechanism of hyperinfection” as accounting for persistence of *S. stercoralis* after leaving an endemic region and highlighted the parasite’s potential risk for veterans of World War II.^15^ The same report differentiated an “autoinfective” cycle of filariform larvae entering perianal skin from a “hyperinfective” cycle in which filariform larvae enter lymphatic channels by penetrating the bowel wall. Inconsistent language continued. A 1958 case report on “overwhelming strongyloides infection” complicated by Gram-negative meningitis variably used the terms “autoinfection” and “hyperinfection” to both describe what Faust formerly dubbed the internal autoinfection cycle.^14^^,^^16^ In a 1966 case report on “overwhelming strongyloidiasis,” the term hyperinfection was supplanted by the term autoinfection, with differentiation between external and internal autoinfection.^17^ A 1967 report discussed a case of “massive strongyloidiasis” related to “autoinfection, caused by the lighting up of an old infection.”^18^

By 1970, there are reports of “hyperinfection syndrome” that feature “massive infestation of the bowel” and an association with Gram-negative bacteremia.^19^ The terms “disseminated strongyloidiasis” or “disseminated strongyloides infestation” were also used in 1970s reports; however, conflation with “hyperinfection syndrome” is apparent.^20^^,^^21^ In recognition of proliferating terms, Grove proposed the terms “uncomplicated strongyloidiasis” and “severe, complicated strongyloidiasis” in his 1989 textbook.^6^ The terms “disseminated strongyloidiasis” and “hyperinfection syndrome” continue to be used in contemporary literature with varying degrees of differentiation between the two.^2^^,^^3^^,^^22^^–^^24^ Isolated reports have also used the term “superinfection” to denote either bacterial superinfection due to larval migration or high parasite burden.^25^^,^^26^

The complexity of the *S. stercoralis* life cycle creates difficulties when condensing its associated disease states into a manageable number of terms. At present, the terms hyperinfection and dissemination convey nearly synonymous forms of strongyloidiasis wherein an accelerated autoinfective cycle leads to an extreme increase in larval forms that drive pathophysiology by cycling between the gastrointestinal tract, lymphatic or blood vessels, and lungs. Hyperinfection refers to a large burden of infection, for example, diagnosed based on detection of larvae in sputum or throughout the bowel by histopathology.^25^ During accelerated autoinfection with dramatically increased parasite burden, the detection of larval or other forms outside these three domains generally defines dissemination. In either disease state, severe bacterial superinfections can occur as the result of migrating larvae and/or gastrointestinal ulcerations. It is important to note that random migration of filariform larvae to ectopic sites and subcutaneous tissue (i.e., *larva currens*) may occur in chronic strongyloidiasis with a normal parasite burden, and this generally does not fall under the term “disseminated strongyloidiasis” because the requisite increase in absolute number of worms is absent.

In the above vignette on *E. coli* bacteremia after high-dose glucocorticoid therapy, distinguishing between hyperinfection and dissemination requires certain data and events to come to clinical attention. For example, development of enterococcal meningitis would be highly suggestive of central nervous system dissemination, but its absence does not exclude the possibility that larval forms migrated well outside the typical circuit of autoinfection. Without histopathological confirmation of dissemination on biopsy specimens, teasing apart hyperinfection from dissemination may not always be possible on clinical grounds. Both terms refer to accelerated autoinfection that dramatically increases the absolute worm burden and has an associated risk of severe secondary bacterial infections. These features of strongyloidiasis may in part explain the confusing mix of terms in the available literature. It is instructive to consider that several authors’ treatment recommendations for strongyloidiasis do not distinguish hyperinfection from dissemination.^3^^,^^23^^,^^27^^,^^28^

Instead of hyperinfection and dissemination, David Grove proposed the terms “uncomplicated strongyloidiasis” and “severe, complicated strongyloidiasis” in his 1989 textbook.^6^ This simple dichotomy captures the clinician’s approach and concern when evaluating patients with strongyloidiasis; however, the broad applicability of the term “severe, complicated strongyloidiasis” is a double-edged sword because it sidesteps the thorny issue of how to characterize a case with high parasite burden when a comprehensive understanding of the distribution and differing tissue burden of parasites is unknowable. For this reason, the terms “hyperinfection” and “dissemination” continue to play functional roles in the nosology of *S. stercoralis*.

Overall, we recommend using the term “hyperinfection” when the net state of immune suppression leads to a dramatically increased *S. stercoralis* worm burden that is not detected outside its typical circuit of the gastrointestinal tract, lymph or blood vessels, and the lung. Hyperinfection can be clinically distinguished from “dissemination” because patients with hyperinfection may be chronically ill rather than critically ill, and secondary bacterial infections are theoretically less frequent. The term “dissemination” presupposes a high worm burden and ought to be used when *S. stercoralis* is clinically detected outside the abovementioned circuit. In contrast to hyperinfection, patients with dissemination are often critically ill, and the detection of disseminating filariform larvae should trigger a broadened work-up for other sites of organ involvement (e.g., lumbar puncture for altered mentation). In addition, empiric antibiotic coverage for enteric flora is often considered in disseminated cases. Thus, the terms “hyperinfection” and “dissemination” are clinically actionable and remain relevant to contemporary practice.
